# The Birth of Bio-data Science: Trends, Expectations, and Applications

**DOI:** 10.1016/j.gpb.2020.01.002

**Published:** 2020-05-16

**Authors:** Wilson Wen Bin Goh, Limsoon Wong

**Affiliations:** *^1^*School of Biological Sciences, Nanyang Technological University, Singapore 637551, Singapore; *^2^*Department of Computer Science, National University of Singapore, Singapore 117417, Singapore

## Components of bio-data science

Biology is becoming increasingly digitized and has now taken on the sheen of a quantitative scientific discipline. A key driving factor is the increasing pervasiveness of high-throughput technological platforms in biological research, allowing millions of data points on genes, proteins, and other biological moieties across thousands of tissues and organisms to be compiled, cleaned, stored, and integrated for the purpose of systematic studies. In this data-rich landscape, it is not an exaggeration to say that the future of biological (and where deployed on clinical samples, biomedical) research lies in strategic maximization of data.

Big bio-data is not a distant fantasy. Not only have we already been living in the age of big bio-data, biological data is also being generated and accrued in an increasingly accelerated manner. Between 1990 and 2003, unraveling the human genome cost approximately $2.7 billion and took several years with many teams involved for completion [1]. By 2016, the same experiment now costs less than $1500 and requires only an afternoon within a single laboratory. Similarly, mapping a single tomato genome initially took an international consortium 5 years [2]; but today, 150 different tomato genomes may be completed within a year [3]. The big bio-data landscape has also spurred the development of big data management systems such as the Expression Atlas [4] and proteomics identification (PRIDE) database [5].

The rise of big bio-data needs to be leveraged upon for understanding diseases and improving health. Problems in the generation, management, analysis, visualization, and interpretation of data should assume a leading role, requiring a paradigm shift in attitude and know-how. Moreover, addressing larger data volumes requires advances in database management platforms and also improved algorithm efficiency. Where large amounts of data are accrued, issues with regard to veracity and complexity also emerge and need to be tackled with more urgency than ever. Traditional disciplines such as bioinformatics and computational biology are now more challenged than ever. In today’s technological landscape, data science and artificial intelligence (AI) have already acted as innovation drivers in areas such as business and finance, where data scientists take helm in converting data into practicable insights instead of working behind the scenes in operations. Examples include AI-driven algorithmic trading and stock recommendation systems in financial technology (fintech) and automated engine design, system maintenance, and robotics in engineering. Given the recent data explosion of and concomitant advances in data science in other disciplines such as business, finance, and computing, we predict that alongside the rapid and voluminous generation of biological data, a new variant of data science, which will specifically address domain-specific issues pertinent to biology, will emerge. We term this variant of data science as “bio-data science (BDS).”

**Figure 1:**
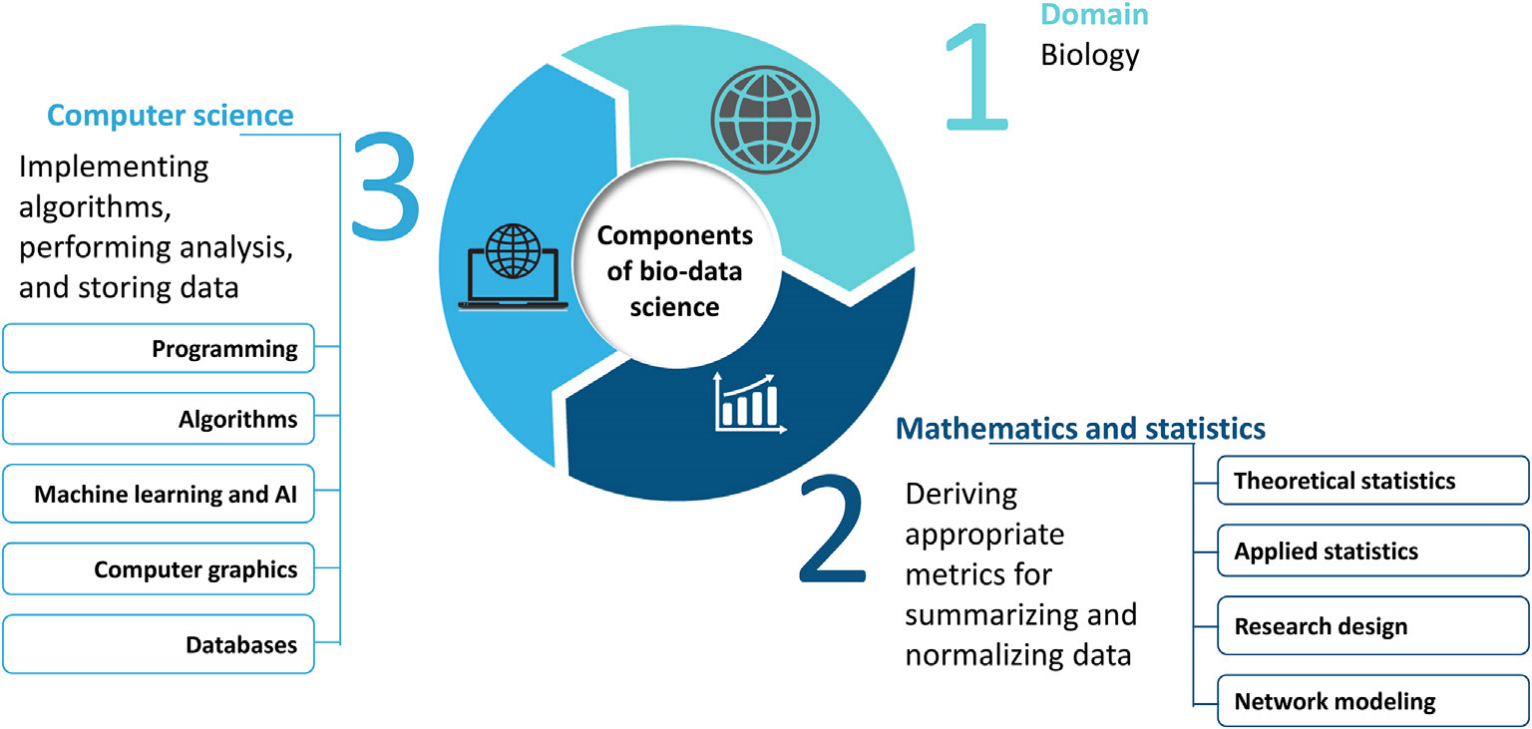
**The core areas of bio-data science** Bio-data science may be split into 3 core areas. The theory is supplied by mathematics and statistics, put into action by computers via computer science, and the biology domain. ML, machine learning; AI, artificial intelligence.

BDS comprises three core disciplinary areas: biology (which constitutes the application domain), computer science, as well as mathematics and statistics (Figure 1). The biology core area is concerned with questions regarding biological origin, such as the cause of a disease or understanding the diagnostic utility of an inferred biomarker. The computer science core area is concerned with devising appropriate algorithms for problem-solving, dealing with repetition (*e.g.*, running the same algorithm on large subsets of data many times over), and resolving data storage issues, especially if the data to be analyzed is large. The mathematics and statistics core area is concerned with issues such as data summarization, normalization, and modeling. Although descriptive and exploratory statistical data analysis is by no means unique to BDS (also being an essential component of biostatistics and, to a lesser degree, bioinformatics), BDS has an added focus on prediction using emerging technology based on applying AI/machine learning (ML) on big data.

Thinking of BDS additively in terms of the disciplinary cores is a mistake. BDS is more than the sum of its parts. Data science is often likened to storytelling with data. And to tell a good story requires one to have in-depth domain knowledge, such that these idiosyncrasies are carefully considered during data interpretation. In other words, BDS requires synergy amongst its disciplinary core areas. To give an example of the importance of domain and synergy with statistics, proteins do not operate independently but rather, as functional units called protein complexes. For a complex to function, its components must be co-expressed tightly, so that the complex can form in the first place. However, when we interpret a matrix of gene or protein expression from a purely statistical viewpoint, we mistakenly assume that each gene or protein operates independent of each other, a fundamental assumption of many statistical tests. This means that when we try to limit false positive rates, we make corrections based on the total number of genes being considered, even though the genes are not independent of each other (*e.g.*, two proteins in a protein complex tend to be correlated in their expression profiles). Assuming independence results in overcorrection, causing loss of statistical power. In such cases, a more reasonable approach would have been to make corrections based on the potential number of protein complexes that can be formed instead [6], [7], [8]. Therefore, the biological domain does not merely create the questions that need to be answered, but it also provides constraints that must be understood and incorporated to create robust models.

We may also categorize BDS by analytical outcomes. Borrowing from Gartner (www.gartner.com), data science outcomes may be categorized into four levels in the order of difficulty and value: descriptive, diagnostic, predictive, and prescriptive. We have summarized these outcomes and levels in Table 1.

**Table 1:** **The four levels of a bio-data science analysis goal or achieved outcome**

**Outcome level**	**Analytical feature and aim**
Descriptive	The simplest form of analytics; Involving reorganization and condensation of data; Using summary statistics to “summarize” the data
Diagnostic	Built on top of descriptive analytics; Likely involving denoising, renormalization, and bias correction; Inferring relationships in data; Aimed to identify key causes
Predictive	Built on top of descriptive and diagnostic analytics; Likely involving the use of clustering and ML techniques (data modeling); Aimed to predict the identity of an unknown entity or determine when a phenomenon will happen (for example, cancer relapse)
Prescriptive	Built on top of descriptive, diagnostic, and predictive analytics; Involving advanced ML and AI techniques (cause-effect modeling); Aimed to influence the occurrence of a phenomenon (If I do this, this will/will not happen); Usually non-straightforward rules for identification

*Note*: ML, machine learning; AI, artificial intelligence.

Currently, most modern-day investigations are at the first two levels. Descriptive analytics is concerned with simple data exploration and data description by plotting basic graphs such as pie charts and line graphs, as well as calculating simple statistics such as mean and median. Diagnostic analytics goes a step further and is concerned with identifying potential underlying causes that can explain why something happens. For example, if the stock market crashes today, we may examine existing data to identify potential causes. It may so happen that a political crisis occurs somewhere else. We know that in general, political uncertainty leads towards economic instability; so, this is a potential explanation, even though it may not in fact, be the correct explanation. The purpose of diagnostic analytics is to attempt, given evidence constraints, to figure out the true cause. To see why it is so hard to determine the true cause in, say, a stock market crash, we only have empirical data showing correlations in the past linking uncertainty and market crashes; it may be just that these two phenomena tend to happen together, that’s all. The more direct way to determine the true cause with certainty is to test for causality; however, it would be unethical and unfeasible to deliberately cause a crisis, just to observe its impact on the stock market.

Predictive analytics is concerned with translating what we currently know, into judgements on future phenomena. Unlike diagnostic analytics, which retrospectively analyzes the possible explanatory causes, predictive analytics goes a step further and attempts to predict the phenomena before it happens. In order to do so, it needs to have a good grasp on the potential causes and appropriate indicators. But this is all it requires, a good grasp on the causes and indicators. It may be able to predict that something will happen; however, without knowing how the causes and indicators actually work together, it is helpless to change what will eventually happen.

Being able to control outcome is the realm of prescriptive analytics. Here, a good grasp on the causes and indicators is not enough. Prescriptive analytics demands that you know how the causes work together, and how changes in specific factors will result in a change consistent with the desired outcome. When working with complex systems, although prescriptive analytics is incredibly difficult to achieve, it is also powerful. Prescriptive analytics requires a deep and detailed understanding of the system. In a complex system where many alternative pathways exist, several factors need to be targeted simultaneously in order to achieve an intended effect. Hence, network modeling in biology has proven to be especially vital for prescriptive analytics [9].

Categorization of BDS by core area or by outcome is useful for theoretical discourse but has otherwise limited practical value. Moreover, in the case of core areas, notions of what should constitute core skills and expertise for data scientists is rapidly evolving. As we enter the “third wave” (at the point of writing), strategic and leadership skills are being increasingly touted as critical areas for enablement and empowerment, which is hardly surprising, as without any charm or charisma, it is difficult to convince other stakeholders to act on advice. As bio-data scientists are probably less concerned with the exigent needs of the business sector, such revisions in the core skill set are useful but nonessential. We do hold the viewpoint that regardless of anyone’s beliefs regarding what should be a disciplinary core area, being an effective bio-data scientist is less about what one knows, than what one does with it. Therefore, emergent skills and behaviors that arise from such divergent multidisciplinary training is more important than the core content (knowledge and skills) themselves. We also cannot emphasize enough that to be an effective bio-data scientist, it is critical to leverage on idiosyncrasies and informative contexts drawn from domain knowledge and use these creatively for problem-solving.

As far as analytical outcome level is concerned, there are also some gray areas. For example, descriptive analytics may also involve denoising and normalization approaches to some extent without the use of any correlation analysis. Also, an intended “prescriptive” analysis may fall short, perhaps due to unresolvable technical errors or other reasons, such that the predictive model cannot generalize and therefore has to be abandoned at the “diagnostic” level.

Ultimately, these divisions and classifications, no matter by disciplinary component or by analytical outcome, are arbitrary.

**Figure 2:**
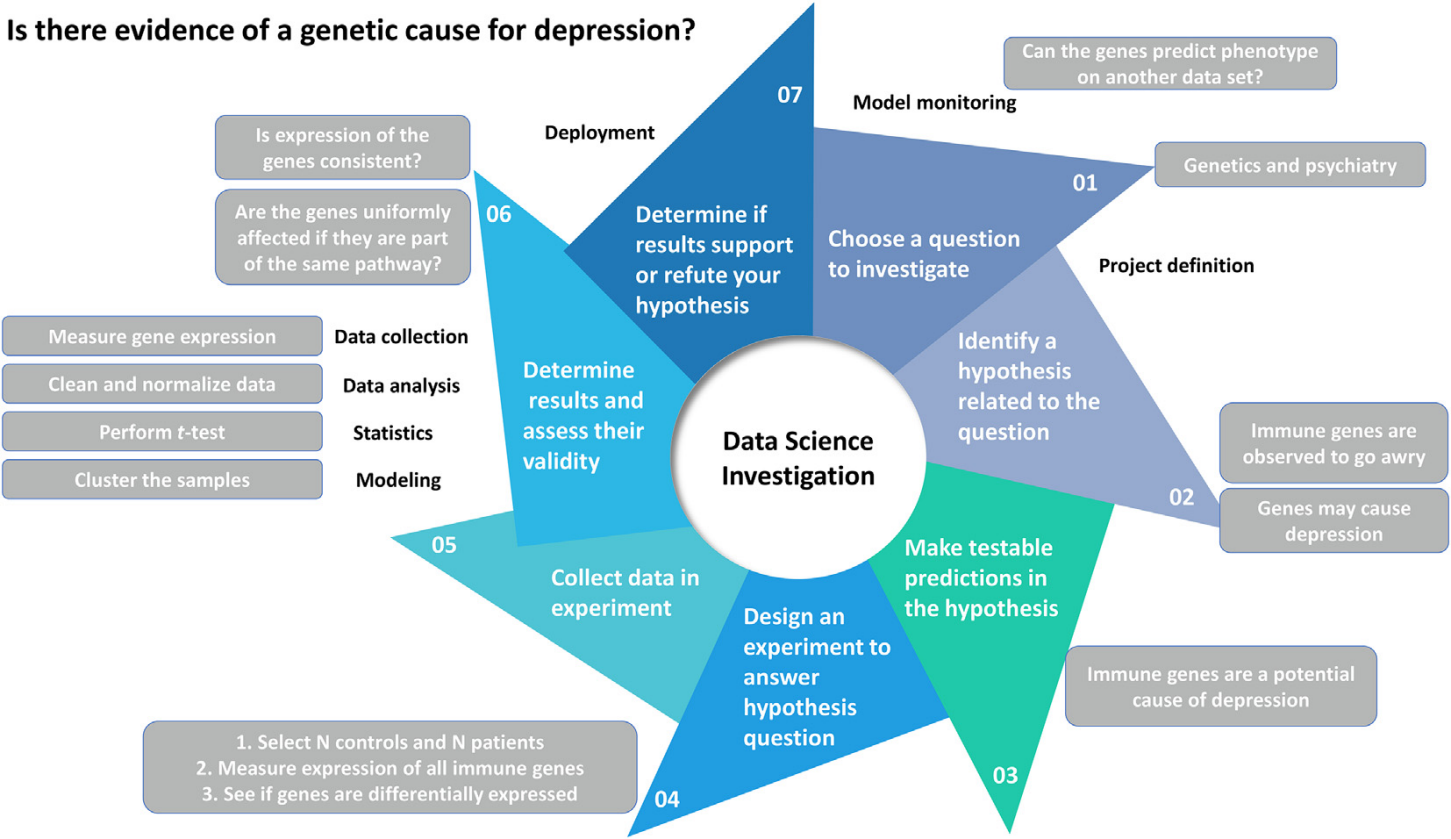
**A bio-data science inquiry requires a well-defined question** Data science is like any other scientific pursuit. It can involve first choosing a question to investigate. We can then scope this question by identifying a relevant hypothesis, which is testable. Appropriate experiments for obtaining data to answer the hypothesis can be then designed and fielded. We then determine the results and assess their validity, that is, whether the data is suitable for answering the research question. Finally, we deploy the model and see if our findings are repeatable.

Despite its seemingly “new” status, BDS is ultimately a science of inquiry, and in this respect, not different from any typical scientific investigation. In the example shown in Figure 2, as a simplified mode of BDS inquiry, we may use the following seven steps to help us answer the question of whether alterations in gene expression correlate meaningfully with mental states. The main difference is that BDS requires strong ability in meaningful data manipulation and analysis, with less emphasis on lower-throughput or underpowered physical experiments.

## Bioinformaticians and computational biologists can be bio-data scientists

We define BDS as the application of data science principles and associated technologies for deriving insights from bio-data. This has important implications for drug development, personalized medicine, automated diagnosis, and health service monitoring systems. Currently, some bioinformaticians, depending on their scope and/or research question, already function as bio-data scientists.

Bioinformatics is the application of information technology (IT) and computer science (CS) to biology. It emerged and evolved in response to the growth of digital biological information, which creates new analytical problems. For example, when full-length DNA or protein sequences became more common, data storage, organization, and representations emerged, paving the way toward pioneering databases such as Dayhoff’s Atlas of protein sequence in 1966 [10]. In the dawn of the Human Genome Project (HGP) and the emergence of DNA-sequencing technologies, it was unnecessarily arduous to identify overlapping DNA fragments by eye. Such tasks are highly repetitive and can be automated by designing and implementing appropriate algorithms. Bioinformatics emerged in a time to provide support for these emerging analytical requirements. Some successes of bioinformatics include the provision of algorithms for assembling a full genome or performing highly intensive annotation tasks, such as marking approximately 10 million single nucleotide polymorphism (SNP) locations in the human genome. Bioinformatics also includes algorithms for noise removal and bias correction. This includes normalization procedures such as robust microarray analysis (RMA) [11] in microarrays, base-calling [12], and gene length-based correction approaches, *e.g.*, transcripts per million (TPM) and reads per kilobase million (RPKM) [13], in RNA sequencing.

Bioinformatics draws upon IT and CS concepts to identify suitable parallels, create reasonable models, and then solve the biological problem. In this respect, bioinformatics acts as a support discipline that solves a technical issue, so that the biologist may move forward in dissection of some biological problems, such as unraveling causal mechanisms that give rise to a phenotype. However, a bioinformatician acting in this respect does not take the lead in generating actionable interventions or building possible explanatory models that lead directly toward understanding the biological problem.

This is not to say that all bioinformaticians do not care about developing models that explain biological phenomena. Certainly, within many laboratories, many bioinformaticians double up to provide explanatory models by collaborating closely with biologists. We regard activities requiring a bioinformatician to translate the digitized data output into biological insight as the realm of computational biology. A bioinformatician can therefore act as a computational biologist.

Both bioinformaticians and computational biologists may act as bio-data scientists, provided they use similar skillsets associated with the data science field. This includes being able to tweak, optimize, and deploy ML and AI technologies, and being well trained in applied statistics. Notably, these are not formal training requirements for computational biologists and bioinformaticians currently.

Computational biologists, bioinformaticians, and bio-data scientists will occupy and share the analytical space in this new digital biology landscape. The distinctions can be muddy, but there is certainly no barrier for a skilled individual to occupy all three professional spaces. Moreover, we do not think that there will be any form of superseding amongst the three professions: bioinformaticians will certainly continue to play important roles as frontline data generators and aggregators. This becomes increasingly important, given the large volumes of data being generated. Computational biologists will evolve, and the biological questions that interest them will change as new possibilities open with the advent of big bio-data. Computational biologists may have already been trained bioinformaticians, and certainly, they may cross the barrier/ divide to leverage on data science, thus becoming bio-data scientists themselves and using the new know-how to create new solutions to their interested biological problems.

Finally, bio-data scientists, no matter self-professed or by professional designation, will emerge as new players. They may be existing purveyors from a bioinformatics or computational biology background. Nonetheless, they may also include new players with non-biological backgrounds such as mathematics, physics, and engineering, or even, pure data science or AI training backgrounds. Just as data scientists are transforming other fields, we foretell the emergence of a new breed of bio-data scientists, who will actively shape and lead the narrative for research and development direction in their chosen biological domains or disease contexts.

## Drivers for BDS

BDS is accelerated by three main drivers: the emergence of big bio-data, the second coming of AI, and a revolution in statistical thinking.

### Emergence of big data in biology

HGP marked the start of numerous large-scale data acquisition initiatives such as the International HapMap Project [14], 1000 Genomes [15], the Cancer Genome Atlas (https://www.cancer.gov/tcga), as well as the recently announced Human Brain Project [16] and the Human Proteome Project [17]. These ambitious initiatives require advances in approaches for data generation, data flow, data storage, data access, and data representation. To address this need, new cloud technologies provide powerful methods for data storage and access beyond the limitations of our local hard drives [18]. Parallel/distributed computing methods such as Hadoop provide powerful ways of performing analysis on the cloud [19].

Today, genotypic data based on DNA and RNA sequences is the major driving force for the evolution of biology into a data science. There are more than 2.7 million samples that are currently available from the Gene Expression Omnibus database (at the point of writing: November 19, 2018). Assuming the size of each file is approximately 1 GB (a very modest estimate), size of these samples can easily add up to the amount of 2.7 petabytes (PB). Improvements in RNA sequencing technologies will accelerate data explosion. For instance, the current Illumina HiSeq X sequencing platform can generate 900 billion nucleotides of raw DNA sequence within 3 days. It is estimated that by 2025, the storage of human genomes alone will require 2–40 exabytes (EB) [20], [21]. Besides genotyping data, other sources of big bio-data are also emerging. These include medical records, phenotyping and trait-based measurements collectively referred to as “phenome,” imaging and microscopy data, as well as network-based information garnered from various interaction-based experiments.

This changing data landscape did not go by unnoticed. In a survey across 704 National Science Foundation investigators, the unanimous response was that biology is awash with big data [22]. Respondents also ranked training on integration of multiple data types, data management and metadata, as well as scaling analysis to cloud/high-performance computing as the three greatest unmet needs critical to advancement in their research fields [22]. It appears that the problem is the growing gap between the accumulation of big data and the limited knowledge of researchers about how to use it effectively [22].

### Second coming of AI

Despite AI being heralded as the technological game changer that will drive the digital economy of the future, this is not the first time such high expectations have been heaped on the AI technology. During the 1970s–1980s, AI was expected to usher in the age of the self-driving car and other technological marvels; these unfortunately did not come to pass, eventually leading to a period known as the “AI winter” [23]. Improvements in AI-based learning platforms, particularly neural networks [24], and newly revitalized paradigms such as deep learning (DL) [25] and reinforcement learning (RL) [26] have created new opportunities and applications.

RL is loosely defined as learning that does not require perfect or large amounts of data. Encapsulated as an AI system, RL is about making appropriate decision and then taking action to maximize reward in a particular situation or acting under specific constraints (*e.g.*, chess playing rules).

DL is loosely defined as architectures that facilitate complex decision-making by modeling AI as neural networks, not unlike the neural connections found in the human brain. DL is compatible with big datasets with high levels of complexities as it aims at learning feature hierarchies from the data, where higher-level features of the hierarchy are formed by composition of lower-level features. We may think of these multi-level features as abstractions, allowing a system to learn complex inputs without necessarily depending completely on pre-defined human-based inputs. DL is gaining great popularity in biological research, with novel applications in proteomics [27], genomics [28], and biomedicine [29], [30], [31].

While there is much anticipated potential that has led to several high-profile tie-ups between IBM’s Watson AI and various pharmaceutical giants (see the section “Trends and expectations for BDS”), it is important to remember that AI and ML techniques are intimately connected, of which the latter is already commonplace in bioinformatics. Algorithms for gene finding based on hidden Markov models (HMMs), *e.g.*, GENSCAN [32], and neural networks for motif finding [33] are just a few notable examples. A key difference between the AI applications of old days, and today’s new applications is scale, wherein AI is expected to identify long-range patterns and perform multi-omics integration across various levels of big bio-data, such as the genome and proteome, thus proposing mechanisms and/or testable targets.

### A revolution in statistical thinking

The field of statistics is undergoing a major transformation. Scientific arguments based solely on *P* values are no longer viewed as sufficiently robust. For example, a replication study across leading psychology journals has revealed that <50% of the studies examined are replicated [34]. Halsey et al demonstrate the instability and variability of *P* values; even as sample size increment and exact replication experiments (EREs) converge on the true effect size, there lacks any concomitant reduction in the variability of *P* values [35]. Halsey et al’s work partially explains the high non-replication rates in Ioannidis’ experiment [34] and warns against the use of the convenient yet ill-founded strategy of claiming conclusive research findings solely on the basis of *P* values, despite it being a commonly accepted practice.

Relatively simple mitigating measures against *P* value instability include using confidence intervals (CIs) [35] (although this viewpoint has also been confronted by van Helden [36]), ranking variables by effect sizes [37], reporting the *P* value replicability or p-rep [38], [39], and performing repeated subsampling on the data to determine if the findings are consistent [40]. There has already been much discussion regarding the nature of *P* values; therefore, we will not elaborate this further.

A very useful, and in our opinion, a more balanced approach is to incorporate Bayesian thinking, when it comes to reasoning about the *P* values. The Bayesian perspective says that instead of only considering the evidence that suggests support for a true effect, we should consider the evidence in totality, which also includes considering the same evidence that suggests support for a non-true effect.

We may express the probability [P(T|e)] for a true effect (T), given some evidence (e). By Bayes’ theorem, the probability is expressed as follows:

(1) $$\text{P}\left(\text{T}|\text{e}\right)=\left[\text{P}\left(\text{T}\right)\times \text{P}\left(\text{e}|\text{T}\right)\right]/\text{P}\left(\text{e}\right)$$

The right hand side is the probability of obtaining a true effect, P(T), which is multiplied by the probability of obtaining some evidence, e, given a true effect, P(e|T), and divided by the probability of observing the evidence, e, independently. We also need to consider the probability [P(−T|e)] for a non-true effect (−T), given the same evidence (e). Accordingly, the probability is expressed via Bayes’s theorem as follows:

(2) $$\text{P}\left(-\text{T}|\text{e}\right)=\left[\text{P}\left(-\text{T}\right)\times \text{P}\left(\text{e}|-\text{T}\right)\right]/\text{P}\left(\text{e}\right)$$

The right hand side is the probability of obtaining a non-true effect, P(−T), which is multiplied by the probability of obtaining some evidence, e, given a non-true effect, P(e|−T), and divided by the probability of observing the evidence, e, independently.

Given some evidence e, we may then calculate the odds of obtaining true effects against non-true effects as follows:

(3) $$\text{P}\left(\text{T}|\text{e}\right)/\text{P}\left(-\text{T}|\text{e}\right)=\left[\left(\text{P}\right)\left(\text{T}\right)\times \text{P}\left(\text{e}|\text{T}\right)\right]/\left[\text{P}\left(-\text{T}\right)\times \text{P}\left(\text{e}|-\text{T}\right)\right]$$

When people observe strong effect (*e.g.*, a significant *P* value) in support of their hypothesis, they will think that there is a true effect. However, they often fail to consider the alternative possibility that a significant *P* value can also arise when there is no true effect. Thus, the Bayesian perspective is more balanced. We can use this perspective in practical settings. For example, when a gene is reported as significantly correlated with a phenotype, we will be less inclined to immediately declare this finding as important without first estimating the likelihood that the same gene will also be reported as significantly correlated, even if it has no true correlation with the phenotype. This perspective can also be usefully extended toward situations beyond “no effect” to situations wherein a significant result is due to a confounder (*e.g.*, batch effects) as well.

A second important and changing statistical perspective is the movement against blind use of centralities such as mean, mode, and median. In symmetrical distributions, the arithmetic mean and median, combined with a sense of the underlying dispersion such as the standard deviation or interquartile range, are generally useful metrics. However, there are many instances wherein the use of centralities is unwarranted and extreme metrics such as minimum and maximum values are actually more useful [41] in situations, including adverse environments where a biological phenomenon is rare [42]. To provide an example, suppose we are interested in examining the optimal configuration for fire resistance, given a fixed number of trees and lakes in a simulation model. The model that provides the maximum number of surviving trees would be the optimal configuration we want. Suppose we simulate the random placement of trees and lakes and return the number of surviving trees each round. In this case, reporting the average values of the models only tells us on average what is the remaining number of trees but is otherwise pointless.

The third perspective is the recognition of the gap between theoretical and applied statistics. The studies of Halsey et al and Ioannidis et al, wherein the former reports *P* value instability leading to the Winner’s curse (the analogy pertains to one winning the lottery out of sheer chance, just as a false positive but spectacular finding also arises due to chance) and the latter shows that >50% of real-world studies in psychology are not reproducible, have demonstrated that theoretical statistical perspectives do not work well in practice [34], [35]. Similarly, in our own practice, we have also found that statistical significance is abundant, due to the presence of confounders and other irrelevant factors [43]. This is also known as the Anna Karenina effect. Such problems are remediable by performing statistical analysis more logically and considering disparities and idiosyncrasies associated with both statistical techniques and data [43].

## Trends and expectations for BDS

The rise of data science, AI, and ML has led toward several high-level collaborations between industry and computing firms, spawned new biotechnology companies, and created new opportunities for advancing scientific discovery.

We list a few examples. In late 2016, pharmaceutical giant company Pfizer announced a collaboration with IBM, involving the use of the latter's Watson AI for immuno-oncological research. In June 2017, GNS Healthcare and Genentech (Roche) announced a collaboration to use the causal ML and simulation platform of GNS Healthcare to power development of novel cancer therapies. In that same month, Novartis also announced a collaboration with IBM Watson to use AI for improving health outcomes in patients with breast cancer. New enterprises are also emerging rapidly. For example, XtalPi is a pharmaceutical technology company that is re-inventing the industry’s approach toward drug R&D with its Intelligent Digital Drug Discovery and Development (ID4) platform, which integrates quantum mechanics, AI, and cloud computing, thus allowing pharmaceutical companies to increase their efficiency, accuracy, and success rates at critical stages of drug R&D. Since 2016, Bayer has been offering money through its grant programs, with clear preference for AI medical startups working on cancer (Turbine) and preventable diseases (xbird).

Besides pharmaceuticals, there are also instances of AI-led advances in biological research. For example, Allen Cell Explorer uses ML to predict stem cell topology based on thousands of images; BenevolentAI and Microsoft Academic AI are learning algorithms that process natural language, formulate new ideas from what they read, and sift through vast chemical libraries, medical databases, and conventionally presented scientific papers to establish connections across knowledge networks.

Biological education is also expected to benefit from the advancement of AI/ML and data science. Smart learning platforms based on adaptive learning models are emerging [44].

## Risks for BDS

**Figure 3:**
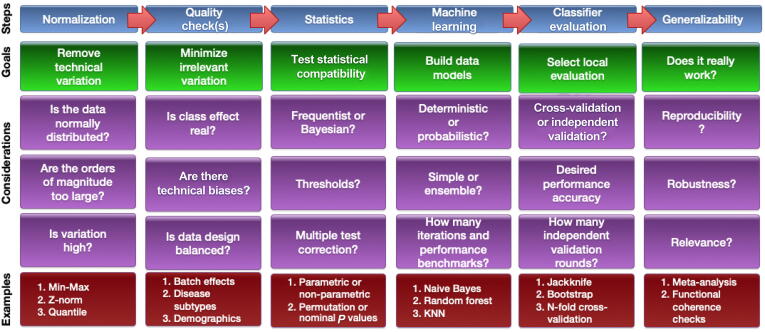
**An analysis pipeline for biomarker analysis using a data-centric approach** While it is foolhardy to propose a one-size-fits-all approach toward biomarker prediction, a workable model may take the form as follows. It can be seen that tools typically associated with data science, such as machine learning, come much later in the pipeline, and are subject to good experimental design and adequate removal of confounding factors from data. A few definitions are provided here for the reader’s convenience: reproducibility – the tendency for an identified signature to be repeated in another independent evaluation; robustness – the tendency for an identified signature to outperform randomly generated feature sets; and relevance – the consistency in terms of a signature with a given phenotype. KNN, K-nearest neighbor.

BDS will be a challenging field, but its difficulties are not necessarily distinct from those of bioinformatics or computational biology. Biological systems are highly complex, while the technological platforms intended for assaying these biological systems are in themselves also highly sophisticated. Moreover, technological instruments developed for measuring biological entities are subject to technical uncertainty, while the components of biological systems change and vary naturally over time. Big bio-data is not a natural solution for such issues, and it presents new difficulties. While big bio-data may facilitate data science endeavors, such as the process of identifying conserved patterns over very large numbers of observations, it may only do so if appropriate analytical pipelines are developed. This task is non-trivial. One may imagine such an analytical pipeline as an end-to-end integration of various approaches, forming an analysis stack starting with data collection and continuing through computational and statistical evaluations toward higher-level biological interpretations and insights. A simplified pipeline for biomarker analysis from high-throughput omics data and the associated key considerations are shown in Figure 3.

Analytical pipelines need to be very flexible and change according to the needs of the research question. Since we lack perfect knowledge, it is also usual to iterate and refine, moving back and forth across several steps, to achieve some sense of optimization and reproducibility. For example, suppose in the normalization step, we find that the use of two different normalization procedures results in very different and non-overlapping differential gene sets. It is possible that the normalization procedure makes erroneous assumptions about the data or that it may have been wrongly implemented. The key considerations shown in Figure 3 are non-exhaustive. The purpose of showing the steps with examples of considerations is to demonstrate that while there is no perfect system or pipeline, given each step, there are many considerations, with each decision point having consequence for the steps that come afterward. We also need to evaluate compatibility issues, such as whether a particular normalization approach works well with a downstream statistical procedure. Other issues include whether a particular procedure might lead toward over-cleaning and overcorrection (problems associated with batch effect correction algorithms [45], [46] and some multiple test correction methods [47]). BDS may be likened to recipe development in the kitchen, requiring multiple rounds of trial-and-error, while keeping a close eye on the intended endpoint or objective. There is no route map or standard operating procedure that guarantees a universally good result. In this regard, BDS is as much an art as it is a science [43], [48].

Suppose we are able to reach our intended analytical objective; it still should not be forgotten that the output is ultimately based on inference. And inferences, when based on massive data wherein we are less able to control heterogeneity and variability, run the risk of generating errors (both false positives and false negatives). These errors in turn lead to overfitting, that is, the predictive models are over-tuned to work well only on the training data but not on future independently generated datasets.

In practice, good research and development should include an accurate evaluation of error rates, and good methods should minimize error rates where practical. However, there is always a trade-off between getting only correct answers (higher false negative rate) and getting all the correct answers (higher false positive rate). Furthermore, estimations of error rates may be off, if the statistical model is a poor fit with the data, for example, using reference models that assume normality of distribution when the data is clearly non-normally distributed.

## Toward a unified BDS curriculum

There are many insertion points into BDS. A computer scientist, statistician, or fintech analyst may enter the field by increasing their biological domain knowledge. A practicing computational biologist and bioinformatician may strengthen their statistical knowledge, learn parallel computing platforms such as Apache Spark or Hadoop, and learn how to use ML and AI implementations such as TensorFlow. Professional training in the BDS landscape will prove highly heterogeneous. It would take more work (and time) for a pure biologist to crossover, as fundamental training in mathematics, statistics, and computing would be required.

**Figure 4:**
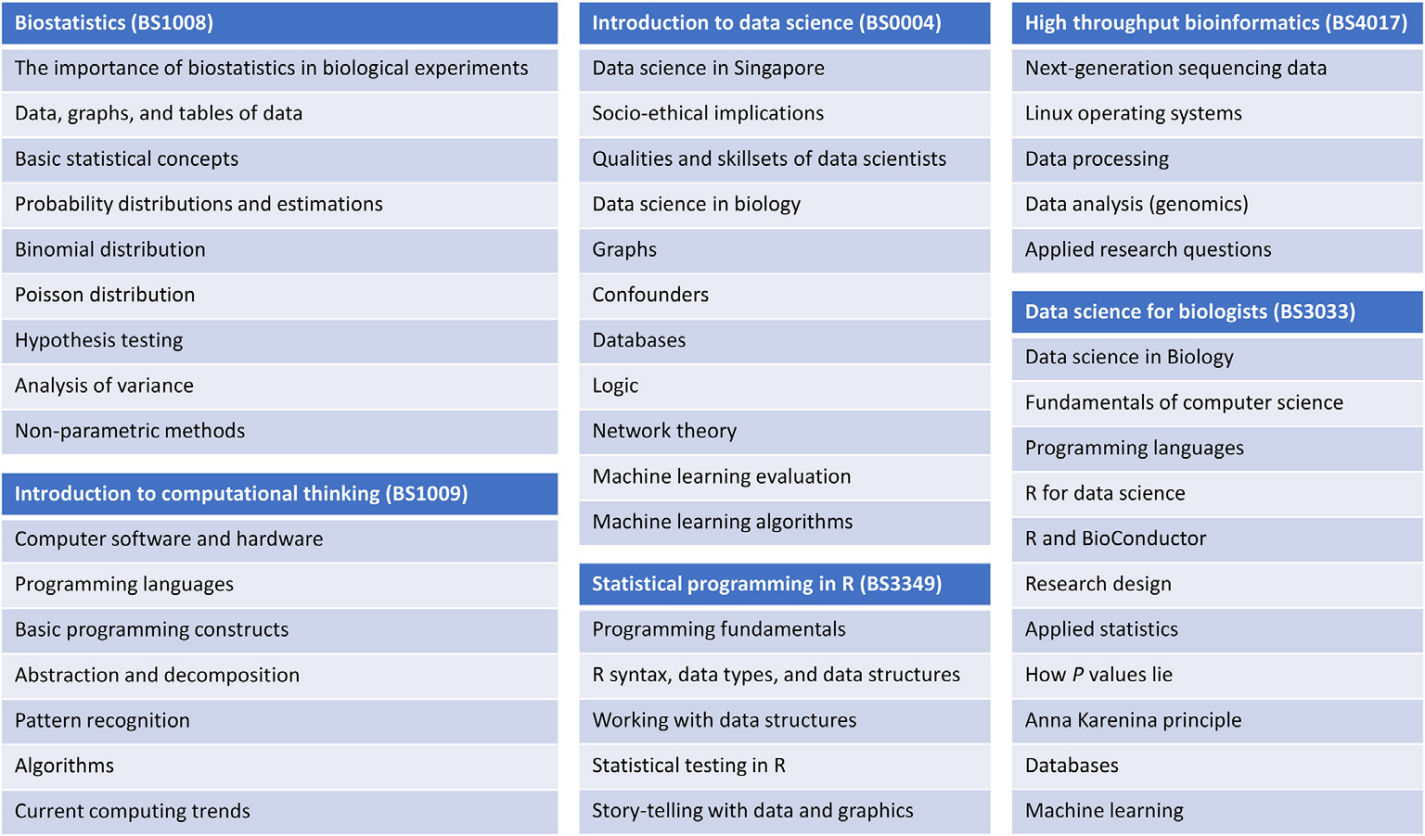
**Current digital literacy offerings in SBS, NTU to facilitate immersion into the bio-data science** SBS currently (at the point of writing: November 19, 2018) offers 2 compulsory data science modules in the form of introduction to computational thinking, and introduction to data science (other modules are electives). The purpose of these modules is to seed interest and also to inform students on the new data-centric paradigms that will likely revolutionize biomedical research in the years to come. SBS, School of Biological Sciences; NTU, Nanyang Technological University.

The increased momentum toward data science has led to education reforms internationally. In recent years, the University of California, Berkeley and Carnegie Mellon University have sought to make digital literacy (basic programming and data science) a core component of all undergraduate education. Where BDS is concerned, the School of Biological Sciences, Nanyang Technological University (NTU), in consultation with other stakeholders, has proposed the following curricula (Figure 4) for BDS. The basic purpose is to equip biological science undergraduates with timely computational thinking and digital literacy skills essential for the modern economy. This set of courses is also meant to provide an insertion point for undergraduates to pursue further training as bio-data scientists.

At the graduate level, a handful of Masters/PhD-level programs using the term BDS have emerged as well. The University of Wisconsin–Madison has launched a pre-doctoral training program and Master’s program in BDS, with emphasis on statistics, mathematics, data visualization, and ML. Other similar and related programs include the Masters of health data science, available in the Faculty of Biology, Medicine and Health, University of Manchester, UK; the Masters of biomedical research (data science), available in the Faculty of Medicine, Imperial College London, UK; and the Masters of biostatistics and data science, available in the Graduate School of Medical Sciences, Cornell University, USA. Beginning from 2020, the School of Biological Sciences, Nanyang Technological University (NTU) will also offer a Masters program in biomedical data science.

We believe what should constitute the core curriculum of BDS is still being formulated and may take several more years before the field matures and stabilizes. We have noticed that the term BDS is now being marketed in some graduate programs. In several cases, besides a name change, the distinction between BDS and bioinformatics/biostatistics is not explicit. While we agree that bioinformatics knowledge is essential in BDS training, it is less clear as to exactly which aspects of bioinformatics are relevant and must be included. The advent of BDS will also drive changes in bioinformatics education, as educators re-examine the course content for timely relevance, and explore areas for synergistic collaboration. Indeed, given the rise of big data, educators are questioning if current bioinformatics curriculum include sufficient components to address this issue. After all, many bioinformatics programs were established before big data became a prominent area of focus.

For biology educators seeking to implement a BDS curriculum, we feel that it is crucial not to just teach programming and employing existing software tools such as TensorFlow [49]. Educational components incorporating abstract, algorithmic, and logical thinking (computational thinking), which are important for problem-solving, are absolutely necessary.

### Some analytical situations requiring BDS

BDS will emerge as a new discipline in light of novel challenges stemming from big bio-data, an increasing recognition of the gulf between applied and theoretical statistics, and expectations heaped upon it given the rise of AI. In this section, we describe some interesting challenges for BDS.

### Creating new perspectives in doing cross-validation right

In our “Turning straw into gold” paper, it is shown that about 50% of randomly generated (and therefore meaningless) gene signatures work well on a given breast cancer survival dataset, with some even outperforming published signatures [50]. On the surface, this would imply rather dramatically that all manuscripts focusing on finding prognostic signatures on breast cancer survival are a waste of effort (and therefore, that all manuscripts with focus on finding such signatures should be rejected without review). Of course, that would be too drastic. However, it does suggest that if we rethink more deeply, even higher stringency should be placed on validation than currently practiced by data mining or ML researchers. In particular, given the observation that a random signature has about 50% chance to be significant in a dataset, more independent datasets must be used to ensure that the observed associations are not due to chance.

Assuming that the datasets are fully independent, we also observed that seven datasets are needed to ensure that a random signature has <1% chance to be universally significant in all seven datasets. This requirement (of seven independent test datasets) is much higher than the common practice of simple cross-validation on a training dataset and a single independent test dataset in the data mining and ML communities. In other words, biology demands higher proof of generalizability.

### Perceived interdependence of datasets in independent validation

In our meta-analysis of various breast cancer datasets, we also observed that the number of independent datasets in which a randomly generated gene signature is significant is not distributed according to the binomial distribution, although the mode of the distribution is preserved and accentuated [50]. This suggests that the independent datasets might not be fully independent despite being collected from different independent groups. Perhaps there are some shared intrinsic population characteristics that confound the random signatures (besides the effects of proliferation-associated genes, which is reportedly a major source of confounding effects). A deeper investigation into the meta-characteristics of these datasets is therefore useful and may reveal the existence of yet, unreported confounders. In other words, while existing ML and AI practitioners may use only one independent validation approach, there are instabilities associated with this extremely crucial step. Just because an independent validation proved positive does not mean that the gene signature is truly good. It could also be because it so happens that the independent validation dataset has some commonalities with the training data, and that therefore, data leakage has occurred.

### Stop to question even when prediction accuracy is good

Suppose we train a neural network W, on a training set and test it on a test set only to get a high accuracy (*e.g.*, 90%). Next, we randomly remove two edges in W to get a new network W′ and train/test it on the same training/testing set as W, it is very likely to get a high accuracy similar to that of W.

Now, suppose we randomly generate lots of new test data and feed these to both W and W′. Although we have no idea what the true class labels on the new test data are, we still can determine whether W and W′ agree on these test data (*i.e.*, W and W′ agree—both predict “yes” or both predict “no,” or W and W′ disagree—one predicts “yes” and the other “no”). It can be observed quite often that W and W′ would drastically disagree on the new test data (with disagreement rates that may be >50% of the new test instances). This means that despite having very similar and common origins, we may have the following findings. (1) W and W′ are drastically different rules/models; (2) a single test dataset is insufficient to validate W and ensure that it is meaningful; and (3) there is often significant sampling gap/bias in a test dataset. A corollary is: (4) it is critical to carefully analyze W to obtain/derive a full explanation of the set of rules it represents and to properly ascertain the biological meaningfulness of these rules.

In short, the ability to achieve a good prediction accuracy may have little to do with the true biological meaning. This is also a major stumbling block when transcending from “predictive” toward “prescriptive” analytical levels. While we offer no direct solution to this problem, it is important to realize that ML and AI are but tools with high tuneability and many performance exceptions. It is therefore important that aspiring bio-data scientists to train hard on logical thinking processes instead of merely relying on feeding massive heaps of data into an algorithmic blackbox. If good results are obtained, the good performance may be misleading. If bad results are obtained, knowing the likely factors to consider and test is crucial, instead of trial-and-error, which may prove daunting when there are many more variables in big data to consider.

## Conclusion

Biology has a golden opportunity to ride on the current data science wave. This will inevitably give rise to a new subfield—BDS. We are beginning to see new initiatives and achievements as a result. We foresee the rise of bio-data scientists as a new breed of specialists who will act as navigators and overseers in directing and leading future innovation from a data-centric/informed perspective.

## Competing interests

The authors have declared no competing interests.
